# Matrix Metalloproteinase Inhibitors and Their Potential Clinical Application in Periodontitis

**DOI:** 10.3390/diseases13090296

**Published:** 2025-09-06

**Authors:** Daniela Mendoza-Juárez, Manuel Sánchez-Gutiérrez, Aleli Julieta Izquierdo-Vega, Eduardo Osiris Madrigal-Santillán, Claudia Velázquez-González, Jeannett Alejandra Izquierdo-Vega

**Affiliations:** 1 Academic Area of Medicine, Institute of Health Sciences, Autonomous University of the State of Hidalgo, Ex-hacienda la Concepción, Tilcuautla 42160, Mexico; 2 Academic Area of Pharmacy, Institute of Health Sciences, Autonomous University of the State of Hidalgo, Ex-Hacienda la Concepción, Tilcuautla 42160, Mexico

**Keywords:** metalloproteinases, metalloproteinase inhibitors, periodontitis

## Abstract

Matrix metalloproteinases (MMPs) are a family of endopeptidases recognized for their involvement in the degradation of the extracellular matrix and their important role in the pathogenesis of periodontitis. This chronic inflammatory condition causes the degradation of dental supporting tissues, resulting in bone loss. In patients with periodontitis, the expression and activation of MMPs, especially MMP-8 and MMP-9, significantly influence tissue degradation. In periodontitis treatment, various natural or synthetic metalloproteinase inhibitors (MMPIs) and antibiotics are used in sub-antimicrobial doses. However, while the evidence supports a role for MMPIs in reducing inflammation, preserving connective tissue, and improving the results of conventional periodontitis treatment, their clinical application is limited. In this review, we summarize MMPIs, their characteristics, and the mechanisms of action that may support their use in the treatment of periodontitis. In conclusion, MMPIs are a therapeutic alternative with great potential in the management of periodontitis, especially when combined with mechanical treatments, although further research is needed to optimize their clinical use.

## 1. Introduction

Periodontitis is a multifactorial chronic inflammatory disease associated with a dysbiotic biofilm and characterized by the degradation of dental supporting tissues, manifesting as loss of clinical attachment, alveolar bone loss, the presence of periodontal pockets, and gingival bleeding [1]. The WHO estimates that 19% of the world’s adult population suffers from this disease, representing the sixth most common chronic condition in the world [2]. The prevalence of periodontitis increases with age, from adolescents to older adults [3], and is associated with a series of genetic, environmental, and local factors, including tobacco use, a high-carbohydrate diet, poor dental hygiene, systemic diseases such as diabetes, and aberrant inflammatory responses to stimuli, and these can act individually or together [2,4,5].

Periodontitis is not only a bacterial infection of the gums, but also the result of an imbalance between the inflammatory response and the host immune system. This immunological imbalance plays a crucial role in disease progression and periodontal tissue destruction [5,6]. An exacerbated host response leads to oral dysbiosis that activates an excessive inflammatory response, resulting in an increase in gingival crevicular fluid rich in collagen-degrading products, immunoglobulins, complement components, cytokines, chemokines, collagen peptides, and products of gingival degradation by matrix metalloproteinases (MMPs) [5].

MMPs are a family of zinc- and calcium-dependent endopeptidases involved in the degradation of extracellular matrix (ECM) components such as collagen, elastin, and fibronectin. They are among the most important ECM proteases. These enzymes activate various growth factors, cell surface receptors, and adhesion molecules [7]. Under physiological conditions, they participate in processes such as embryogenesis, morphogenesis, angiogenesis, tissue remodeling, and wound repair [8] by degrading and promoting the turnover of collagens, elastin, and gelatin [9], as well as promoting cell survival, exhibiting antitumor action, and regulating hormone receptors [7]. They can also modify cytokines, growth factors, and adhesion molecules, suggesting complex functions of chemical mediators.

**Figure 1:**
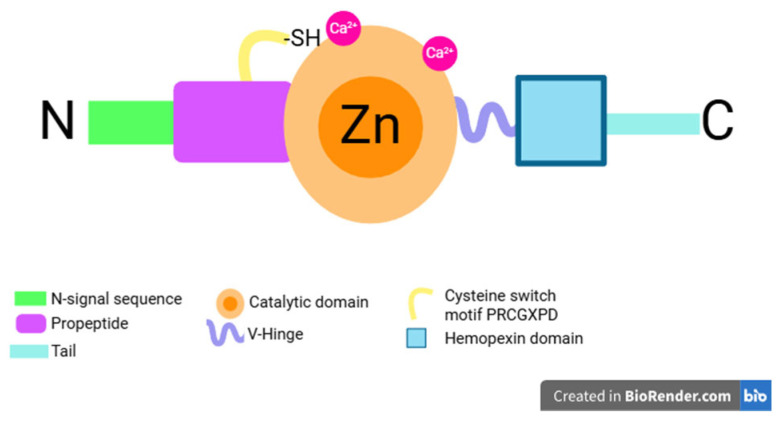
Common structure of MMPs. Created in BioRender. Mendoza-Juárez, D (2025) https://app.biorender.com/illustrations/684c975b4a8a56ff43ee9a39.

There is a common structure to most MMPs. They comprise a pro-peptide with an N-terminal signaling peptide end that varies in length, a catalytic domain containing a zinc ion, a linker peptide known as the hinge region, a hemopexin domain, and a tail [9,10] (Figure 1). In humans, MMPs have 23 members encoded by two distinct genes, which can be distinguished into two main types: MMPs secreted into the extracellular matrix as proenzymes, and membrane-bound MMPs that bind to the cell membrane [11]. They are classified according to their substrate specificity and homology into 6 groups: collagenases (MMP-1, -8, -13, -18), gelatinases (MMP-2, -9), stromelysins (MMP-3, -10, -11), matrilysins (MMP-7, -26) and membrane-type metalloproteinases (MT-MMP) (MMP-14, -15, -16, -24, -17, -25). In addition, MMP-12 (metalloelastase), MMP-20 (smealisin), MMP-28, MMP-22, and MMP-23 are individually uncategorized [12,13,14].

Membrane-type matrix metalloproteinases (MT-MMPs) belong to a subgroup of the matrix metalloproteinase (MMP) family, distinguished by their location in the cell membrane. These enzymes play roles in the restructuring of the extracellular matrix and the regulation of physiological and pathological processes. Currently, six MT-MMPs have been identified, divided into two categories, namely membrane-type (MT-1, 2, 3, and 5 MMPs), which have an intracellular domain and a transmembrane region that anchors them to the plasma membrane, and glycosylphosphatidylinositol-anchored type (MT-4 and 6 MMPs), which are linked to the membrane via glycosylphosphatidylinositol. MT-MMPs are involved in ECM degradation, the activation of other MMPs, and the regulation of cellular processes. MT1-MMP plays a crucial role in osteoclastic activity, facilitating bone matrix degradation during bone restoration [15]. This review aimed to provide an overview of the various types of MMP inhibitors (MMPI), their properties, and mechanisms of action that could be evaluated and applied in the treatment of periodontitis.

## 2. Methods

In this review, a systematic search of the scientific literature, including reviews and clinical trials, was conducted in various databases, such as PubMed and Scopus, up to June 2025. The search strategy employed a combination of keywords related to metalloproteinase inhibitors/periodontitis. The search strings used were “metalloproteinases”, “matrix metalloproteinases”, “metalloproteinase inhibitors”, “periodontitis”, and “periodontal inflammation”, using Boolean operators. Included were articles with population, in vitro, and animal studies, reviews, and meta-analyses. Articles written in languages other than English were excluded.

## 3. Pathophysiology of Periodontitis and MMPs

Periodontitis is associated with an imbalance between MMPs and tissue inhibitors of metalloproteinase, collagenases, and gelatinases. These are the main enzymes involved in periodontitis because the main component of the ECM is collagen type I, present in the periodontal ligament [16]. Collagenases are the main MMPs associated with tissue degeneration, dividing the components of the ECM. These include MMP-1, -8, and -13, degrading interstitial collagen I, II, and III, respectively [16,17]. The gelatinase MMP-9 also plays an important role in periodontitis [17].

MMPs are produced by various cells and tissues such as connective tissue, vascular tissue, endothelial tissue, fibroblasts, osteoblasts, endothelial cells, macrophages, and neutrophils [9]. In addition to physical and chemical agents, chaotropic agents, and LPS, proinflammatory cytokines like IL-1β or TNF-α bind to transcription factors to induce MMP synthesis in the form of pre-pro-MMPs and pro-MMPs zymogens that are activated by endopeptidases and other active MMPs or macrobiotic proteases [11,18]. Non-proteolytic oxidative activation of MMPs is essential in periodontal inflammation. Reactive oxygen species (ROS) activate MMPs in periodontal tissues through direct enzymatic oxidation and by indirect mechanisms, such as the secretion of myeloperoxidase due to neutrophil degranulation stimulated by periodontal-pathogenic bacteria. This generates oxidants such as hypochlorous acid that degrades connective tissue [16,19,20] and activates pro-MMP-8 [21,22] and pro-MMP-9 [22]. MMPs degrade different types of collagen, including types I, II, III, IV, V, VI, VII, VIII, IX, X, and XIV, as well as entactin, fibronectin, tenascin, laminin, myelin basic protein, and vitronectin [9].

Different types of MMPs are expressed in the oral cavity. MMP-8 is one of the main MMPs responsible for the irreversible destruction of periodontal tissues [11], and its high levels in gingival crevicular fluid correlate with the severity of periodontitis [23], accompanied by symptoms such as loss of attachment [17,24], periodontal pocket depth, and bleeding on probing, as well as high plaque index [17]. MMP-8 is present in odontoblasts and is the most important active collagenase in dentin [25]. It facilitates the migration of neutrophils to periodontal tissues by degrading tissues that form the ECM, and it degrades collagen, fibronectin, aggrecan, fibrinogen, bradykinin, angiotensin 1, serpins, and substance P [16,26]. It is characterized by its ability to degrade collagen types I and III, which causes periodontal destruction not observed in normal gingival tissue remodeling [27]. MMP-8 is synthesized in the bone marrow during polymorphonuclear cell development, remaining in zymogen form as pro-MMP-8 within neutrophil granules [28,29]. It is secreted by neutrophils, macrophages, T cells, plasma cells, endothelial cells, and keratinocytes [26] through the action of mediators such as IL-1β, IL-8, TNF-α, the complement system, fibrin-degraded products, and granulocyte colony-stimulating factor [16,25]. It is regulated by transforming growth factor-β, stromelysins, trypsin-2, cathepsin G, and IL-1β [30]. It is subsequently activated by the action of MMP-3 and MMP-10 [31], bacterial proteases present in the dental biofilm [32], free radicals, trypsins, and serine proteases [33]. It is also activated in the extracellular space through the cysteine switch system [34], comprising a proteolytic elimination on interaction of the cysteine residue of the prodomain and the zinc ion of the catalytic site [19]. This pro-domain harbors a conserved motif, usually with the sequence PRCGXPD, in which the cysteine residue plays a fundamental role [9] (Figure 1). The inhibition of MMP-8 enzymatic activity is affected by MMPIs and macroglobulin-α2 [35].

Active MMP-8 (aMMP-8) can be measured to detect periodontal tissue damage, according to previous research. This helps guide treatment and suggests that adding aMMP-8 testing to the periodontitis classification system could improve diagnostic accuracy [36,37]. According to Deng et al., (2021), gingivitis and periodontitis can be distinguished from each other by elevated levels of aMMP-8, but not by elevated levels of total or latent MMP-8. A higher risk of clinical attachment loss and disease progression may be indicated by elevated aMMP-8 levels. Early, non-invasive detection of active periodontal tissue destruction is made possible by measuring aMMP-8, which also has important ramifications for forecasting the course of the disease, enabling treatment monitoring, and improving clinical interventions [38]. Interestingly, prediabetes is associated with elevated levels of aMMP-8 using the PerioSafe/ORALyzer lateral flow immunoassay, reflecting an oral inflammatory response and faster progression of periodontitis. This highlights the importance of early detection of prediabetes in periodontal clinics to improve the management of both conditions [39]. More recent evidence-based studies propose that controlling the periodontal situation with appropriate and timely therapy could improve glycemic control in diabetic patients [40]. According to recent meta-analyses, MMP-8 and aMMP-8 are useful diagnostic biomarkers for both periodontitis and gingivitis [41,42]. Although one study found no significant differences between MMP-8 and aMMP-8, methodological improvements and standardization are necessary for their clinical use. Enhancing diagnostic methods across various sample types is important for improving patient diagnosis, care, and outcomes [41].

Another MMP present in the progression of inflammation in patients with periodontitis is MMP-9, which degrades collagen types IV, V, and XI, as well as proteoglycans and elastin [16]. It is expressed and secreted as a zymogen in endothelial cells, leukocytes, neutrophils, and macrophages [43], with polymorphonuclear cells being the main source. It has been identified at high levels in epithelial and gingival junction cells and connective tissue in advanced stages of periodontitis [11,44]. MMP-9 is synthesized within neutrophils during granulocyte differentiation in the bone marrow, and is regulated by transcription factors E-26 (Ets), NF-kB, activator protein 1 (AP-1), specificity protein 1 (Sp-1), serum amyloid A activating factor (SAF-1), and TNF-α [44]. It is activated by disruption of the zinc interaction with the catalytic domain, as well as by other MMPs [11,44]. Overexpression of MMP-9 activates the expression of osteoclasts by removing collagen from the bone, initiating bone resorption [45]. In addition, cathepsin K activates pro-MMP-9 when osteoclastic activity presents an acidic environment, which causes a decomposition of minerals, exposing collagen fibers and beginning their degradation [46,47]. Its presence is related to the severity of periodontitis and is decreased after periodontal treatment [48].

In periodontitis, the accumulation of plaque with bacteria initiates a chronic inflammatory response that induces the overexpression of various MMPs through secretion or activation. Increased MMP-8 levels are associated with bacteria such as *Fusobacterium nucleatum* [49], *Tannerella forsythia*, and *Treponema denticola* [50], which is why MMP-8 is considered an accurate biomarker for the diagnosis of periodontitis [51]. A decrease in MMP-8 concentrations in crevicular fluid has been observed after periodontal scaling and root planing treatment [22,52,53]. *Treponema denticola* present in biofilm activates the secretion of MMPs [54] through the secretion of proteases that activate MMP-2, causing fibronectin fragmentation, apoptosis induction, and suppression of osteoblast differentiation [16]. MMP-2 is also activated by *Porphyromonas gingivalis*, which increases monocyte migration and activates the expression of MMP-9, destroying periodontal tissues [53].

## 4. Types of MMP Inhibitors (MMPIs)

**Figure 2:**
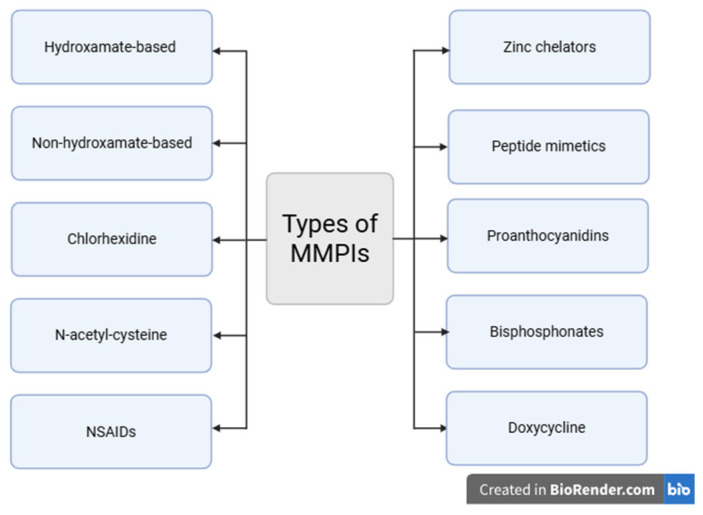
Types of MMPIs. Created in BioRender. Mendoza-Juárez, D. (2025). https://app.biorender.com/illustrations/684ab6e5e637f8636f465232.

Although several MMPIs are known, limited information exists regarding their application in periodontitis, whether in experimental or population studies. This article summarizes the MMPIs, their characteristics, and their mechanisms of action, which may support their clinical use in treating periodontitis. The different MMPI types discussed below, with their mechanisms of action, are summarized in Table 1, and Figure 2.

Because excessive MMP activity causes excessive ECM degradation, these enzymes can be considered therapeutic targets of MMP inhibitors [11]. Hydroxamate-based inhibitors, for example, are molecules with structures based on collagen that imitate the natural peptide substrate of the selected MMP. They contain a group that chelates the catalytic zinc ion, reducing the contribution of the rest of the compound in the inhibitor-enzyme binding process, conferring a broad spectrum of inhibition [8,81,82]. New generation hydroxamate-based MMP inhibitors are more specific, reducing adverse effects. Their development has helped identify the molecular substructures related to the presence or absence of biological activity [81,83]. However, their use is limited by metabolic inactivation of the hydroxamate group and side effects like musculoskeletal pain, which can be caused by their inhibition of other metalloproteinases. Despite these disadvantages, they are attractive due to their high potency as MMPIs [81].

Non-hydroxamate-based inhibitors have been designed to prevent metabolic inactivation and unselective metal chelation of other MMPs. Carboxylates, sulfonamides, and phosphates are examples of non-hydroxamate-based inhibitors created to circumvent the drawbacks of first-generation hydroxamate-based inhibitors. By providing stability and selectivity, these substances can inhibit MMP without the negative side effects of hydroxamates. Some of these inhibitors have also demonstrated improved binding capacity and efficacy by targeting sites other than the MMP catalytic site [81]. Alternative binding sites have also been targeted in inhibition therapy [84], and antibody-based therapies have been developed that manifest high selectivity towards the anchored membrane of the selected MMPs [85].

In periodontitis treatment, adjuvants are used that act as MMPIs, such as bisphosphonates, which prevent MMP production by inhibiting the activity of osteoclasts, exerting a chelating effect on catalytic ions [23]. Another group of compounds, proanthocyanidins (polyphenols), acts by inhibiting the production and activity of MMPs. These compounds inactivate more than 90% of the recombination of MMP-2, MMP-8, and MMP-9 by binding to zinc and calcium ions, inhibiting their catalytic activity [23,86]. Chlorhexidine, the most widely used adjuvant in the treatment of periodontitis, acts as a non-specific MMPI through chelation of calcium and zinc cations, inhibiting MMP-2, MMP-8, and MMP-9 [55,87].

On the other hand, controlling the biofilm associated with peri-implantitis treatments (an inflammatory complication affecting the tissues surrounding dental implants) is a considerable challenge. Despite treatment, a systematic review of clinical trials revealed the presence of *Prevotella intermedia*, *Fusobacterium nucleatum*, and *Peptostreptococcus micros* in submucosal biofilms. However, after treatment, *Prevotella intermedia*, *Tannerella forsythia*, *Treponema denticola*, and *Porphyromonas gingivalis* were detected. This was likely due to the persistence of bacterial invaders and the existence of unresolved pathologies that favored anaerobiosis and dysbiosis [88]. MMP-8 is one of the biomarkers used in the identification of peri-implantitis [89], and a recent meta-analysis of 276 patients found that patients with peri-implantitis had significantly higher MMP-8 levels than patients with healthy implants [90].

## 5. Clinical Application of MMPIs in Periodontitis

### 5.1. Zinc Chelators

MMPs, composed of a variety of proteases, use metal ions to bind to the substrate and to polarize water molecules to carry out hydrolytic reactions. MMPs are among the human enzymes that employ zinc ions for this function. Molecules that block zinc metalloproteinases bear a chemical moiety that replaces the zinc-coordinating water molecule in the catalytic site, deactivating the zinc ion. Phosphinate (PO_2_), carboxylate (COO), thiolate (S), and hydroxamic acid HONH-CO are effective zinc-binding moieties, leading to potent inhibition of zinc metalloproteinases.

TPA (tri-2-pyridinemethylamine) inhibited MMP-14 with a Ki of 1.2 µM, whereas compound Zn148 showed effective inhibition of both pseudolysine (PLN), a zinc metalloproteinase secreted by the bacterium Pseudomonas aeruginosa, and MMP-14, with a Ki of 5 µM. Furthermore, compounds such as dipicolylamine have been shown to increase the rate of substrate cleavage by MMP-14 by enhancing the interaction of the substrate with the enzyme binding site, suggesting complex effects on enzyme activity [56].

Zinc niflumate is a zinc complex of niflumic acid, a nonsteroidal anti-inflammatory drug (NSAID). This compound exhibited selective cytotoxic effects on endometriotic cells, inhibiting cell proliferation and metalloproteinase activity, suggesting its potential as a therapeutic agent in the treatment of endometriosis and other related disorders. In endometrial cell lines, 10 µM of zinc niflumate inhibits the activity of the metalloproteinases MMP-2 and MMP-9, altering gene expression and reducing proteolytic activity, which could be related to the regulation of signaling pathways such as MAPK and COX-2 [57].

Thiolutin is a zinc chelator activated by reduction to its dithiol form. It exhibits antibiotic properties and inhibits zinc-dependent JAB1/MPN/Mov34 metalloenzyme (JAMM) domain metalloproteinases, at concentrations ranging from 0.53 to 2.5 µM, which are responsible for protein deubiquitination. Thiolutin may have the potential to inhibit other extracellular MMPs and angiotensin-converting enzymes (ACE). This is attributed to its ability to chelate zinc, but its efficacy in living cells may be limited because thiolutin is activated intracellularly [58].

The efficacy of zinc inhibitors has been assessed in other experimental models. In experimental autoimmune encephalomyelitis, 1H10 (a chemical inhibitor of AMP-activated protein kinase (AMPK) that also acts as a weak zinc chelator, with a CI50 of 10 µM) significantly reduced the activation of MMP-9 in mice. In addition, a decrease in demyelination, microglia activation, infiltration of immune cells, and disruption of the blood–brain barrier was detected, suggesting that 1H10 reduces the severity of experimental encephalomyelitis [59].

While zinc chelators represent a specific treatment for periodontitis control, their clinical adoption as MMPIs has proven difficult due to their limited selectivity and bioavailability. Furthermore, dose-limiting side effects and inadequate trial designs were reported during clinical trials of these MMPIs. The evaluation of topical zinc chelating inhibitors for the relief of periodontitis presents several challenges, including leaching by saliva and gingival fluid, which can affect the therapeutic concentration, specificity, and local cytotoxicity to fibroblasts and keratinocytes. Furthermore, zinc chelator inhibitors of specific MMPs must present specific structural characteristics. MMP-12 inhibitors, for example, require a hydrophobic group occupying the S1’ space, a zinc-binding motif to chelate the catalytic Zn ion, and a flexible hydrogen bonding region between them to interact with the catalytic amino acid residues and the Ω loop [91]. Patents related to MMPIs have recently been filed for diseases such as cancer; however, MMPIs could also be considered for inflammatory periodontal diseases [92].

### 5.2. Bisphosphonates

Bisphosphonates are non-biodegradable analogs of pyrophosphate that can release calcium ions and have a high affinity for hydroxyapatite crystals. They are used as low-toxicity therapeutic agents to slow the progression of bone loss and prevent radicular resorption by inhibiting osteoclast activity and phagocytosis of bone crystals containing bisphosphonates [60,61]. Clodronate, a drug used to treat hypercalcemia, has demonstrated inhibitory capacity on the activity of MMP-1, MMP-3, MMP-8, and MMP-9 by acting as a cation-chelator [61,62]. Zoledronate, another drug used to treat hypercalcemia, suppresses MMP-2 and MMP-9 expression in PC3 prostate cancer cells, indicating that it is involved in the Ras/Raf/ERK and PI3K/AKT pathways, in addition to Discoin Domain Receptors (DDR) signaling [63]. The mechanism of action by which bisphosphonates inhibit these enzymes involves the chelation of calcium and zinc cations [62]. Tiludronate, another bisphosphonate, has also been observed to upregulate tissue inhibitors of MMPs in human periodontal ligament cells [64]. Other synthetic MMPIs contain a bisphosphonate group linked through a sulfonamide to an aryl moiety that binds to a specific segment of the enzymes, promoting selective MMP inhibition [93,94]. De Colli et al. compared the effects of these new bisphosphonate MMP inhibitors with those of zoledronate in human gingival fibroblasts exposed to *Porphyromonas gingivalis* LPS. Compared to zoledronate, a preservation of membrane integrity was observed with compounds (4-nitro-phenylsulfonylamino)methyl-1,1-bisphosphonic acid and (biphenyl-4-sulfonylamino)methyl-1,1-bisphosphonic acid. A significant decrease in MMP-9 and MMP-14 expression was observed for all three compounds [95]. However, only the new bisphosphonate MMP inhibitors present the advantage of preserving membrane integrity.

A randomized, double-blind, placebo study over 12 months was conducted in patients with moderate to severe periodontitis, excluding those with diseases affecting bone metabolism. Patients received nonsurgical periodontal treatment, consisting of scaling and root planing and periodontal maintenance every 3 months. Patients were randomized into groups receiving bisphosphonate (alendronate 10 mg/day or risedronate 5 mg/day) or placebo, plus calcium citrate (1000 mg/day) and vitamin D3 (400 IU/day). Those in the bisphosphonate groups showed significant improvement in clinical attachment level, reduced probing depth, and decreased bleeding on probing, so bisphosphonate therapy may be an effective adjunctive treatment for preserving periodontal bone mass [96].

Bisphosphonates offer a potential treatment for periodontitis by optimizing clinical parameters and promoting bone regeneration. However, their use requires careful evaluation due to associated risks, such as the adverse effect of osteonecrosis after prolonged treatment lasting 6 to 12 months. Further clinical and experimental studies are needed to assess the incidence of osteonecrosis related to bisphosphonate treatment and to evaluate the effectiveness of short-course bisphosphonate treatment [97].

### 5.3. Peptide Mimetics

Peptide mimetics are compounds created to closely resemble the structure and functionality of natural peptides to maximize characteristics like biological activity, stability, and bioavailability. They have been used to stimulate and regulate cells in various physiological processes, such as inflammatory response, metabolism, cell signaling, and tannin regeneration, as well as to create new medications that mimic peptides linked to certain disorders [98].

Collagen mimetic peptides (CMP) are short, single-chain peptides that mimic the structure of collagen, specifically with a repeating sequence of prolines and glycines, such as Pro-Pro-Gly. They have a high affinity for damaged collagen, intercalating at breaks in collagen strands and helping to restore its original structure. These peptides have been used in treatments to repair damaged ocular tissue, promoting healing and improving collagen fibril alignment after injury. In a recent study, tissue stiffness measurements were conducted using atomic force microscopy (AFM) on rat optic nerve head sections. The tissues were treated with MMP-1 for 30 min, followed by a 60 min treatment with the collagen-mimetic peptide CMP-3. Treatment with MMP-1 significantly decreased tissue stiffness in the peripapillary space. Following treatment with CMP-3, improved tissue stiffness restoration, a significant decrease in collagen fragmentation in the sclera and glial lamina, and improved collagen fibril alignment were observed, suggesting its potential to repair damaged collagen in ocular tissues [99].

In another study, the effect of the Ac2-26 peptide mimetic of the annexin A1 protein was evaluated in an in vitro experimental study using HaCaT human keratinocyte cells and a D551 human fibroblast line. Annexin A1 (AnxA1) is a member of the annexin family and has demonstrated strong anti-inflammatory properties in acute, chronic, and systemic inflammation. Treatment with the Ac2-26 peptide showed inhibition of metalloproteases MMP-1 and MMP-8, assessed by Western blot analysis. In addition, an increase in the level of COL1A1 (gene encoding type I collagen) was observed, suggesting a decrease in collagen degradation. The authors concluded that treatment with the Ac2-26 peptide has anti-inflammatory effects and promotes collagen synthesis, suggesting its potential as a cosmetic treatment for skin conditions related to inflammation and aging [65]. In the same context, annexin A1 protein mimetic peptide Ab2-26 was evaluated against TNF-α-induced damage in CHON-001 chondrocytes by assessing cellular senescence and expression levels of MMP-13 and (ADAMTS)-4 (a disintegrin and metalloproteinase with thrombospondin motifs). Ab2-26 restored TNF-α-induced increases in p53 and p16 gene and protein expression in a dose-dependent manner. Furthermore, it was shown that TNF-α produced elevations in MMP-13 and ADAMTS-4 mRNA and protein levels, which were reduced by Ab2-26 in a dose-dependent manner [100].

6KApoEp is an apolipoprotein E (ApoE) peptide mimetic designed to mimic the functions of ApoE in the nervous system. In an experimental study, after inducing hemorrhage, different doses of 6KApoEp were administered to evaluate its effect on blood–brain barrier permeability and neurological function. Neurological scores were measured using the Longa method, blood–brain barrier permeability was assessed by Evan’s blue content in brain tissue, and levels of MMP-9 and other proteins were analyzed by immunohistochemistry. 6KApoEp reduced MMP-9 expression by activating the LRP1 receptor, which inhibits the CypA/NF-κB/MMP-9 signaling pathway. This activation reduces inflammation and the degradation of tight junction proteins, thereby improving the integrity of the blood–brain barrier. Consequently, after intracerebral hemorrhage, the presence of MMP-9 in the affected area decreased [66].

The effect of peptide mimetics on wound healing was demonstrated in an experimental study in mice using wound biopsies. Wounds were treated with different formulations, including platelet-derived growth factor (PDGF)-loaded cogels and cogels containing PDGF polyplexes with collagen-mimicking peptide (CMP) modifications in various percentages, and were evaluated over 14 days to observe healing and other histological parameters. The inclusion of the CMP in the PDGF polyplex-loaded cogels resulted in increased collagen deposition, with a 24% greater rate of wound closure compared to PDGF cogels without CMP. Furthermore, cogels with 20% CMP modification exhibited collagen expression similar to that of healthy skin, enhancing the ratio of type I to type III collagen. These results indicate that CMP improves wound healing by facilitating the controlled release of PDGF and by promoting fibroblast activity [101].

Regarding the potential of peptide mimetics in treating periodontitis, a synthetic cationic antimicrobial peptide, Nal-P-113, has been shown to inhibit and kill periodontal bacteria in a planktonic state (bacteria suspended in a liquid medium, such as saliva, and not adhered to any surface) at concentrations less than 320 μg/mL. This peptide also inhibits biofilm formation and eradicates polymicrobial biofilms at 1280 μg/mL. The mechanism by which Nal-P-113 inhibits and kills periodontal bacteria may involve pore formation within the bacteria’s cytoplasmic membranes and permeabilization, leading to bacterial death [102].

The influence of annexin A1 (ANXA1)-mediated formylpeptide receptor-2 (FPR2) on periodontal pathology was studied in a rodent model of periodontitis and human periodontal ligament cells. ANXA1-FPR2 signaling influenced periodontal tissue destruction. WRW4, a selective antagonist of FPR2 signaling, increased alveolar bone resorption and osteoclast counts, while Ab2-26 reduced these effects. In human cells, the FPR2 antagonist increased the secretion of IL-8 and other factors by silencing ANXA1 and FPR2. The results suggest that ANXA1-FPR2 plays a role in regulating inflammation in periodontal disease [67]. Different peptide mimetics, such as Dhvar4 and Nal-P-113, bacterial adherence regulators (BARs), and D-enantiomeric peptides, have shown antimicrobial activity, inhibiting adhesion and eradicating resistant biofilms. Specific targeted antimicrobial peptides (STAMPs) with a broad-spectrum antimicrobial peptide domain have also been developed, their design resulting in the elimination of pathogenic bacteria without affecting commensal microorganisms [68]. A notable example of a STAMP is highlighted in a systematic review. STAMP C16G2 is an effective sealant against dental caries, with selective activity against *Streptococcus mutans*, and represents a significant advance in reducing oral biofilm and preventing caries, promoting a non-cariogenic oral environment. Randomized clinical trials are needed to evaluate the clinical utility of C16G2 and its efficacy as a routine self-care practice [69]. Peptide mimetics with antimicrobial and anti-inflammatory properties are a novel treatment option for periodontitis, but preclinical research is necessary to verify their efficacy and safety in humans.

### 5.4. N-Acetylcysteine (NAC)

A precursor of reduced glutathione, NAC is a synthetic derivative of the endogenous amino acid L-cysteine, known for its use as an antidote to acetaminophen poisoning. Following oral administration, NAC is quickly absorbed in the small intestine and converted to cysteine, which the liver uses to produce glutathione (GSH). Due to its intestinal metabolism, high hepatic first-pass metabolism, and rapid cellular diffusion and conversion to GSH, NAC has a low oral bioavailability [103]. NAC has potent anti-inflammatory and antioxidant properties, and it is extensively used in the treatment of digestive, cardiovascular, respiratory, neurologic, and psychiatric disorders, as well as chronic pain [103,104,105,106,107,108].

Regarding the effect of NAC on MMPs, MMP-9 is involved in mediating neuropathic pain through increased activity and expression in dorsal root ganglia following nerve injury. MMP-9 is also involved in degenerative and proinflammatory mechanisms that exacerbate neuropathic pain. Intraperitoneal administration of NAC at a dose of 150 mg/kg significantly inhibited MMP-9 activity in dorsal root ganglia and dorsal horn segments of the spinal cord in rats. NAC administration resulted in a significant reduction in remifentanil-induced hyperalgesia, suggesting its efficacy in modulating neuropathic pain through MMP-9 inhibition [109]. Another related finding highlights that NAC inhibits the activation of the MMP-9/RAGE (receptor for advanced glycation end products) pathway, thereby preventing oxidative damage and decreased integrity of parvalbumin interneurons. In patients with early psychosis receiving a dose of 900 mg/L NAC during a 6-month clinical trial, NAC treatment reduced plasma RAGE levels, which was associated with increased GABA levels in the prefrontal cortex and improved processing speed. This suggests that NAC targets the pathological mechanisms mediating MMP-9/RAGE-related oxidative stress [70]. Additionally, it has been shown that vitamin E, NAC, and other MMPIs have anti-inflammatory and antioxidant properties. In combination with vitamin E, NAC contributed to a reduction in the exposure of eye and liver tissues to aflatoxin B1 (AFB1), while increasing exposure in other tissues, suggesting that these compounds may influence metalloproteinase activity and toxin distribution in the body [110]. 

Currently, there is limited information to evaluate the efficacy of NAC in periodontal tissue. Recently, however, the properties of NAC have been applied in the dental field. Nanosilver-based hydrogels containing NAC showed antibacterial and antibiofilm properties, inhibiting *Acinetobacter baumannii* and *Pseudomonas aeruginosa* at concentrations of 20–40 μM [111]. Antioxidative therapy is regarded as a promising treatment for periodontitis since oxidative stress is the primary cause of the disease in the early periodontal microenvironment. In a recent study, NAC was used to create novel red fluorescent carbonized polymer dots (NAC-CPDs) that demonstrated good compatibility and efficacy in scavenging reactive oxygen species (ROS). In mice with periodontitis, NAC-CPDs reduced bone loss by promoting bone cell development and targeting the alveolar bone. By modifying the Keap1/Nrf2 pathway, they may offer a new approach to treat periodontitis [71].

NAC polysulfide (NAC-S2) was also shown to have a beneficial effect on periodontitis in a mouse model. NAC-S2 was administered intraperitoneally (120 mmol/kg) for one week before inducing periodontitis. Macrophages derived from bone marrow and gingival tissue were isolated and cultured. In mice with periodontitis, NAC-S2 decreased the expression of proinflammatory cytokines (TNF-α, IL-6, and IL-1β), reduced the distance between the cementoenamel junction and the bone crest, and alleviated TLR4/NF-κB-mediated inflammation [72]. While NAC is a well-known antioxidant that inhibits MMPs in other models, these studies emphasize the significance of antioxidant therapy in periodontitis. Taken together, these properties support its use in the treatment of periodontitis.

### 5.5. Nonsteroidal Anti-Inflammatory Drugs (NSAIDs)

NSAIDs are medications used to treat pain and inflammatory conditions, primarily by inhibiting cyclooxygenase/prostaglandin-endoperoxide synthase 1 and 2 [112]. The combination of tetracycline with NSAIDs has been reported to inhibit the activity of MMPs [113,114,115], and Lee et al. showed that the use of doxycycline with a low dose of flurbiprofen (50 mg/kg) in chronic periodontitis significantly decreased the levels of MMP-2 and MMP-9 [114]. The effectiveness of NSAIDs in combination with tetracycline is based on increasing blood flow at the site of injury, increasing the local supply of tetracycline [115]. A further study reported inhibition of MMP-9 in corneal lesions in an avian model following treatment with diclofenac sodium and ketorolac [116], while another reported decreased MMP-13 expression in a murine osteoarthritis model after treatment with naproxen [73]. Sadowski et al. demonstrated a significant decrease in the activity and expression of MMP-1 in chondrocytes stimulated with IL-1 after treatment with indomethacin, naproxen, and meloxicam, as well as a decrease in MMP-3 after treatment with meloxicam [74]. A decrease in MMP-1 following treatment with diclofenac potassium has also been observed in a murine orthodontic model [117].

On the other hand, naproxen has been reported to increase the production of pro-MMPs-1, -3, and -9 [118]. Furthermore, cyclooxygenase inhibition has been shown to increase MMP-1 expression in fibroblast-like synoviocytes through inhibition of prostaglandin E-mediated signal-regulated kinase (ERK)-dependent MMP-1 production [119]. Ketorolac and diclofenac sodium have also been reported to induce the expression of MMP-1, MMP-2, and MMP-8 [120], and ibuprofen increases the expression of MMP-1, MMP-8, MMP-9, and MMP-13 in a dose-dependent manner [75].

### 5.6. Doxycycline

An antibiotic belonging to the tetracycline class, doxycycline is a broad-spectrum bacteriostatic agent that is synthetically derived from oxytetracycline, a naturally occurring tetracycline produced by *Streptomyces* species bacteria [121]. The Food and Drug Administration (FDA) has approved doxycycline for the treatment of Lyme disease, ophthalmic infections, anthrax, acute intestinal amebiasis, severe acne, respiratory tract infections, bacterial infections, rickettsial infections, malaria, and traveler’s diarrhea [122,123,124,125,126]. Doxycycline has also been investigated as a treatment for specific cancers on the basis of evidence that it can induce apoptosis and inhibit cell invasion and proliferation [127,128,129].

The detrimental effects of MMP overexpression in chronic lesions, the function of MMPs and their inhibitors in the wound healing process, and the potential of topical doxycycline as a synthetic inhibitor to support tissue regeneration have all been previously established [130,131]. However, there is conflicting evidence regarding the outcomes of doxycycline treatment of periodontitis. Most clinical studies and even meta-analyses have not shown relevant results on clinical attachment levels or probing depth. This could be explained by bacterial resistance, as patients with chronic periodontitis are often resistant to doxycycline, reducing its efficacy. The clinical studies also had limitations, including metabolic dysregulation in diabetic patients, failure to consider other drugs that could influence outcomes, and a high dropout rate [132,133,134].

One study assessed the effects of subantimicrobial doxycycline treatment in patients with stage 2 type 2 diabetes and grade B periodontitis. One group of patients received scaling and root planing (SRP) only, and the other received SRP plus doxycycline. In patients with SRP and a subantimicrobial doxycycline dose of 20 mg/day for three months, a significant reduction was observed in probing depth, gingival index, and gingival crevicular fluid MMP-9 and MMP-13 levels, and improvement was seen in the clinical indices of gingival index and plaque index [135]. In a double-blind, placebo-controlled clinical trial, 128 postmenopausal women diagnosed with osteopenia and moderate to advanced periodontitis received doxycycline or a placebo for 2 years. Treatment with doxycycline significantly reduced MMP-8 levels in gingival crevicular fluid, decreasing the MMP-8 activity associated with collagen degradation. It also showed a reduction in a biomarker of bone resorption, supporting a positive effect on reducing collagen degradation [136]. In a third example, the effects of subantimicrobial doxycycline treatment over 24 months on the microbial flora were evaluated in a double-blind, placebo-controlled clinical trial involving osteopenic women with periodontal disease. The treatment had no detectable effects on the microbial flora, and there was no evidence of cross-resistance or multi-antibiotic resistance [137]. In another randomized, double-blind, placebo-controlled clinical trial, 174 patients with chronic periodontitis received doxycycline (20 mg, administered twice daily) as adjunctive therapy with SRP for 3 months. A reduction in clinical indices (probing depth and bleeding on probing) was observed. A significant reduction was also noted in 3-nitrotyrosine levels, and significant correlations were found between the clinical indices and staining showing nitrosative stress in gingival tissue, indicating that a reduction in nitrosative stress is associated with improvements in periodontal health [138].

In a study evaluating the efficacy of an 8.5% nanostructured doxycycline gel in a rat model of periodontal disease, the effect of doxycycline was evident in preventing alveolar bone loss. Experimental periodontitis was induced by placing a ligature around the neck of the rat’s left maxillary second molar. Topical doxycycline gel (1 g) was applied to the ligated areas three times daily for 11 days, and the efficacy of the gel was assessed by histopathological analysis and atomic force microscopy (AFM). Alveolar bone loss was also measured. Treatment with the doxycycline gel significantly reduced alveolar bone loss, and surface roughness analysis showed that periodontal structures were rougher and better maintained their integrity [139].

In an in vitro study, the proliferation and osteogenic capacity of periodontal stem cells (1 µg/mL) were assessed with doxycycline, which decreased IL-17-induced migration and MMP-2 expression while stimulating osteogenic differentiation and affecting mitochondrial respiration in PDLSCs [140]. Previously, Golub et al. demonstrated different mechanisms of action for tetracyclines at subantimicrobial doses and non-antimicrobial modified tetracyclines in the inhibition of degradation of collagen-rich tissues, including bone resorption. These mechanisms included zinc- and calcium-dependent inhibition of MMPs, inhibition of ROS independent of cation binding by non-antibiotic tetracyclines, inhibition of serine proteases such as elastase, decreased expression of inflammatory cytokines, nitric oxide, and phospholipase A2, and collagen synthesis, osteoblast activity, and bone formation [141,142]. Currently, Periostat is an FDA-approved drug containing a non-antibiotic dose of doxycycline for the treatment of periodontitis, illustrating its safety and efficacy as an adjunct to oral hygiene [143].

Similarly, “host modulation therapy”, a concept introduced in 1980, is recommended for the treatment of periodontitis. This therapy employs strategies that modulate the host immune response to mitigate inflammation and tissue damage in diseases such as periodontitis, thus facilitating the treatment of microbial biofilms [143]. Another strategy to prevent bacterial resistance in the treatment of periodontitis is the use of chemically modified tetracyclines that act as host modulators. In these molecules, tetracycline is modified by removing the C4 dimethylamino group responsible for its antibiotic activity [144].

While adverse reactions such as phototoxicity have been reported with doxycycline and demethylchlortetracycline, which could cause sunburn in patients with certain conditions, a lower risk of phototoxicity is associated with monocycline and lymecycline. Photosensitization reactions to tetracyclines vary according to factors like drug concentration in the skin, skin type, and radiation properties. Regarding toxicity mechanisms, the formation of toxic photoproducts, covalent photobonding of skin molecules, energy transfer, and free radical production have been proposed [76]. An example of a modified tetracycline is incyclinide, which is under evaluation as a host-modulating therapy for chronic periodontitis. This drug acts by reducing tissue degradation and promoting periodontal regeneration, and patients treated with incyclinide showed significantly improved clinical attachment levels. Larger, randomized, placebo-controlled trials are needed to confirm these findings and to establish long-term safety and efficacy [145].

### 5.7. Proanthocyanidins (PACs)

PACs are natural compounds classified as flavonoid polyphenols. They are condensed tannins generated by the combination of flavan-3-ol units such as catechin and epicatechin. Present in the fruits, seeds, bark, and leaves of many plants, well-known sources of PACs include grapes, blueberries, pomegranates, and green tea. PACs have been highlighted for their antioxidant, antibacterial, antiviral, anti-inflammatory, cardioprotective, neuroprotective, and anticancer properties, as well as for protecting and strengthening collagen and elastin [146].

In the field of dentistry, polyphenols such as resveratrol (10 mg/mL) and myricetin (0.2 mg/mL) have demonstrated beneficial effects on the durability of the resin-dentin bond by modifying collagen and inhibiting MMP activity, as evaluated in an in vitro model of human dentin [147]. In a further study using human gingival fibroblasts and osteoblasts in an in vitro model of a dental implant surface, pretreatment with PACs, flavonoids from grape seed extract, and synthetic naringenin was evaluated in terms of the synthesis of MMPs, gelatinases, and their tissue inhibitors, and the gelatinolytic activity of MMPs. The flavonoids controlled the induced gelatinolytic effects, negatively regulating the synthesis and activity of MMPs in fibroblasts and osteoblasts. In the case of osteoblasts, the tissue inhibitors were positively regulated by the flavonoids [77]. Cranberry PACs have potential in the development of host modulation strategies in periodontitis. In vitro research conducted on differentiated keratinocytes and macrophages demonstrated that cranberry PACs decreased MMP production in a concentration-dependent manner, reducing the catalytic activity of MMP-1 and MMP-9. This inhibition was linked to a reduction in NF-κB p65 activity. At 31.25 μg/mL, cranberry PACs decreased growth by 23.83% and biofilm formation by 93.98% [78].

Blueberry PACs (500 μg/mL) have been shown to inhibit the growth of *Aggregatibacter actinomycetemcomitans*, a microorganism that plays an important role in localized aggressive periodontitis and biofilm formation. Moreover, the secretion of proinflammatory cytokines IL-1β, TNF-α, IL-6, and IL-8 is stimulated by the LPS of *A. actinomycetemcomitans*. The PACs also inhibited the secretion of the metalloproteinases MMP-3 and MMP-9 that actively participate in the destruction of oral tissue in periodontitis, and prevented the activation of the NF-κB signaling pathway that plays a key role in inflammatory processes [79].

The use of PACs to improve resin-dentin bonding has also been studied, both in etch-and-rinse adhesives and self-etch adhesives. PACs can crosslink the dentin collagen matrix, thus improving the mechanical properties of the exposed collagen, and can dehydrate the fibrils, creating a better collagen substrate for hybridization. These properties are important for achieving a biostable resin-dentin hybrid interface, which is essential for improvements in current adhesive dentistry [148].

Alkimavičienė et al. studied the effect of PACs in minimally invasive non-surgical therapy in patients with stage III-IV periodontitis. Collagen hydrogels containing PACs were applied to the subgingival region and, after 8 weeks of treatment, clinical periodontal parameters were evaluated. The PAC hydrogels caused a significant decrease in pocket probing depth and an increase in clinical attachment level in periodontal pockets compared to the collagen hydrogel alone. Furthermore, the addition of PACs caused a significant reduction in MMP-3 levels in saliva after 8 weeks of treatment. The concentration of MMP-3 in saliva reflects periodontal health status, so this outcome demonstrates that PACs provide additional beneficial effects on clinical and immunological parameters in the treatment of periodontitis [80].

### 5.8. Chlorhexidine

Chlorhexidine gluconate is a broad-spectrum antimicrobial agent used in dentistry, primarily as an antiseptic mouthwash to prevent oral biofilm buildup and as an adjuvant treatment for periodontal disease [149]. Its antimicrobial action is dose-dependent, with bacteriostatic activity at low concentrations (0.02–0.06%) and bactericidal activity at high concentrations (>0.12%) [150]. An increase in MMPs in the gingival crevicular fluid is observed in advanced stages of periodontal disease [23,48]. Gendron et al. demonstrated in vitro the inhibition of MMP-8 activity at a chlorhexidine concentration of 0.01 and 0.02%, and the inhibition of MMP-9 and MMP-2 at concentrations of 0.03%, through a mechanism involving chelation and interaction with the sulfhydryl groups and/or cysteines present in the active sites of the MMPs [55].

In restorative dentistry, adhesive systems are used that, over time, lose their bonding to the dentin tissue through hydrolysis of the collagen matrix of the hybrid layer and through degradation of the hydrophilic polymers of the adhesive systems [151]. Therefore, preservation of the collagen matrix is an approach to improve the durability of the bond of these systems to dentin [152]. Certain MMPs in dentin are responsible for the degradation of the bonding interface [153], and chlorhexidine is used as an MMP inhibitor [55]. Chlorhexidine is also used as an endodontic irrigant, reducing collagen degradation and improving bond durability [154]. Retana-Lobo et al. reported a decrease in MMP-2 and MMP-9 levels in the lumens of dentin tubules of the roots of extracted human teeth treated with chlorhexidine [155]. This was attributed to a calcium chelating mechanism that affects the requirement of metal ions in the catalytic activity of MMPs [156].

The identification of reliable biological markers that demonstrate enzymatic activity is crucial for monitoring the efficacy of MMPIs. Various research indicated that salivary levels of MMP-8 and MMP-9, regulated by tissue inhibitor of matrix metalloproteinases-1 (TIMP-1), are useful for diagnosing periodontal disease [157,158,159]. A recent meta-analysis questions the usefulness of TIMP-1 as a biomarker for periodontal monitoring, as no significant differences were found in saliva or gingival crevicular fluid in patients with periodontitis. Additionally, TIMP-1 can be influenced by smoking, obesity, osteoporosis, and rheumatoid arthritis [160]. For the evaluation of inflammatory periodontal disease and monitoring the efficacy of localized periodontal therapy, saliva is a simple and non-invasive sample to obtain. As previously reported, activity levels of the salivary biomarkers MMP-8, MMP-9, and IL-1β have been linked to periodontitis. However, standardized sample processing protocols are required before ELISA or zymographic methods to ensure reproducibility and sensitivity.

## 6. Conclusions

MMPs have great potential in the treatment of periodontitis due to their ability to slow the degradation of connective and bone tissue. They are also involved in the degradation of the extracellular matrix during periodontal inflammation, so MMP inhibition can reduce disease progression and provide dental support in treatments complementary to conventional mechanical therapy, such as scaling and root planning. However, further experimental studies and clinical trials are needed to evaluate less-explored natural and synthetic compounds that have shown potential to improve the treatment outcomes of periodontitis.

## Figures and Tables

**Table 1:** Mechanisms of action of MMPI.

MMPI	Action Mechanism	Ref.
Chlorhexidine	Ion chelation, interaction with sulfhydryl and cysteine groups in the active site of MMP-2, MMP-8, and MMP-9.	[55]
Zinc Chelators	Inhibition of zinc-dependent metalloenzyme by replacing the coordinates of the water molecule with zinc at the catalytic site.	[56]
Zinc niflumate	Inhibition of MMP-2 and MMP-9, related to the regulation of signaling pathways such as MAPK and COX-2.	[57]
Thiolutin	Inhibition of MMP-2 and MMP-9, activated intracellularly in dithiol form, inhibits JAMM metalloenzymes (deubiquitinases).	[58]
1H10	Zinc chelator and AMPK inhibitor; MMP-9 inhibitor.	[59]
Bisphosphonates	Calcium and zinc chelator; high affinity for hydroxyapatite crystals; inhibits osteoclast and MMP activity.	[60]
Clodronate	It acts as a cation-chelator. Inhibitor of MMP-1, -2, -3, -7, -8, -9, -12, -13, -14.	[61,62]
Zoledronate	Inhibition of MMP-2/-9 expression, related to the Ras/Raf/ERK and PI3K/AKT pathways and DDR tyrosine kinase receptors.	[63]
Tiludronate	Up-regulates tissue inhibitors of MMP in periodontal ligament cells.	[64]
Peptide mimetic Ac2-26 (annexin a1 mimetic peptide)	Inhibition of MMP-1 and MMP-8 promotes the synthesis of collagen type I (COL1A1), and has an anti-inflammatory effect. Reduces the expression of MMP-13 and ADAMTS-4 induced by TNF-α, which are involved in cellular senescence.	[65]
6kapoep (apolipoprotein E peptide mimetic	Decreases MMP-9 expression by activating the low-density lipoprotein receptor-associated protein-1 (LRP1 receptor); inhibits the Cyclophilin A/NF-κB pathway.	[66]
Annexin A1 (ANAXA1)	Anti-inflammatory effect through the FPR2 receptor, reducing periodontal inflammation and bone resorption.	[67]
Synthetic cationic antimicrobial peptide (NAL-p-113)	Permeabilize bacterial cytoplasmic membranes, preventing biofilm formation.	[68]
Specific targeted antimicrobial peptides (STAMPs)	Pore formation in bacterial membranes.	[69]
N-Acetyl-Cysteine (NAC)	Inhibition of MMP-9/RAGE (receptor for advanced glycation end products) pathway activation in a rat model of neuropathic pain and patients with psychosis.	[70]
	Modulation of antioxidant pathways (Keap1/Nrf2) and promotion of osteogenesis in a mouse model of periodontitis.	[71]
	Inhibition of inflammation mediated by the TLR4/NF-κB signaling pathway and proinflammatory cytokines in an experimental mouse model of periodontitis.	[72]
NSAIDs		
Naproxen	Decreased MMP-13 expression in osteoarthritis models; regulation of inflammation.	[73]
Naproxen, Indomethacin, and Meloxicam	Inhibition of MMP-1 and MMP-3 expression and activity in IL-1-stimulated chondrocytes.	[74]
Ibuprofen	MMP-1, MMP-8, MMP-9, and MMP-13 expression is increased in a dose-dependent manner.	[75]
Doxycycline	Decreased MMP expression and activity. Immunomodulatory and anti-inflammatory effects, stimulation of tissue regeneration. Photosensitivity has been reported as an adverse reaction.	[76]
Proanthocyanidins (PACs)		
Synthetic naringenin	Increases the synthesis of natural MMP inhibitors.	[77]
Cranberry PACs	Inhibitor of MMP-1, -9, inhibition of the NF-κB signaling pathway, and decreases in biofilm formation.	[78]
Blueberry PACs	Reduction in inflammation and bacterial biofilm, as well as inhibition of the NF-κB signaling pathway.	[79]
Collagen hydrogels-PACs	Decreased MMP-3 in the saliva of patients with periodontitis.	[80]
